# Reduction in protein absorption on ophthalmic lenses by PEGDA bulk modification of silicone acrylate-based formulation

**DOI:** 10.1007/s40204-019-00119-x

**Published:** 2019-08-14

**Authors:** Fahimeh Askari, Mojgan Zandi, Parvin Shokrolahi, Mitra Hashemi Tabatabaei, Elnaz Hajirasoliha

**Affiliations:** 1 Polymer Science Department, Iran Polymer and Petrochemical Institute, Tehran, 1497713115 Iran; 2 Biomaterials Department, Iran Polymer and Petrochemical Institute, Tehran, 1497713115 Iran; 3 Publication Department, Iran Polymer and Petrochemical Institute, Tehran, 1497713115 Iran

**Keywords:** Ophtalmic lens, Protein absorption, Silicone acrylate, Poly(ethylene glycol) diacrylate

## Abstract

The absorption of protein and formation of biofilms on the surface of ophthalmic lenses is one of the factors that destroy their useful performance by causing severe visual impairment, inflammation, dryness and ultimate eye discomfort. Therefore, eye lenses need to be resilient to protein absorption, which is one of the opacity factors in minimizing protein absorption on the lenses. The purpose of this study was to investigate and reduce sediment biotransformation on the surface of the semi-hardened lens based on acrylate by bulk-free radical polymerization method. In this respect, the effect of poly(ethylene glycol) diacrylate (PEGDA) with two different molecular weights of 200 and 600 g/mol on the surface roughness, protein absorption, and hydrophilicity of the lenses were studied. The surface hardness of the lenses, on shore D scale, was measured using a durometer hardness test. The presence of higher molecular weight of PEGDA hydrophilic polymeric monomers reduced the hardness of the lenses. The effect of introducing PEGDA, with two molecular weights, into lens fabrication formulations was studied with respect to their water content parameters and hydrophilicity. The presence of a crosslinker such as poly(ethylene glycol) diacrylates, at two different molecular weights, increased the water content and hydrophilicity of the produced lenses. Surface roughness is associated with the formation of bio-film and accumulation of microorganisms on the surface. Due to the roughness of the lens surface developed in this research, the lenses containing PEGDA 600 exhibited less roughness compared to that of PEGDA 200, which could also affect the absorption of protein. Therefore, according to the results of protein absorption test, the PEGDA 600 lenses showed lower protein absorption, which could be due to their high degree of water absorption and hydrophilicity.

## Introduction

One of the common problems in the ophthalmology is opacification of the hard and soft contact lenses. Acrylic-based hydrogels as the main component of contact lens formulations should provide certain physical and chemical properties such as good transparency, excellent permeability (especially oxygen permeability), dimensional stability, hydraulic permeability, and protein and cell repellence, as well as good mechanical properties and biocompatibility with the cornea and eye environment. The main source of opacification could be cell-repellent and protein-repellent depending on the type of materials in lens formulation (Bozukova 2007).

Currently, hydrogel ophthalmic lenses based on a wide variety of different acrylic monomers including 2-hydroxyethyl-methacrylate, acrylamide, acrylic acid, ethylene glycol diamethacrylate, ethylhexyl methacrylate, methyl acrylate, and silicon acrylic monomers were developed (Muter 2015; Musgrave and Fang 2019).

Silicone acrylic-based hydrogels have advantages such as high oxygen permeability and high flexibility, but hydrophobic nature of this group of lenses leads to very low wettability and high protein deposition rates (Santos et al. 2007). To overcome this problem, most hydrogel silicone lenses need to be modified to reduce the hydrophobic nature of the silicon components, as well as decrease the protein deposition on the surface of the lens. To improve the wettability of the hydrogel silicon lenses a few methods such as plasma modifications and copolymerization have been developed (Chen et al. 2018). To enhance wettability and address natural hydrophobicity of the silicone, methacrylic acid is added. Altogether, the addition of hydrophilic monomers to silicone lens formulations, increase hydrophilicity and plays an important role in preventing sedimentation and biofilm formation on the surface of the lenses (Lin and Svitova 2010). Physical and chemical properties of the lens such as water content, refractive index, hardness, mechanical strength and oxygen permeability can be aptly controlled by selection of the appropriate acrylic monomers (Marc et al. 2018).

So far, a couple of oxygen-permeable hydrogel silicone materials have been commercially available with oxygen transmissibility per thickness (*Dk*/*t*) in the range of 65–175 × 10^−9^. These lenses may account for about 30% of all global soft contact lenses (Efron and Maldonado-Codina 2017).

During their use, the tear components, including lipids, mucins and proteins, precipitate on the lenses which lead to discomfort, reduced vision and inflammatory reactions. Protein deposition and biofilm formation on the lens surface is one of the reasons that reduce the lens’s effectiveness. Moreover, it provides a substrate for the accumulation and growth of bacteria. By increasing the amount of deposition, patient’s vision and wearing duration of the lens are reduced.

Despite various treatment options that are available, we believe prevention is the best cure, and choosing the most biocompatible lens is an essential prevention measure. Biocompatibility refers to materials that can function well with respect to the surrounding tissues. Many factors which affect the lens biocompatibility can be manipulated by determining factors such as structural design and material selections (Huang et al. 2016).

This is mainly due to the improvement in surface chemistry by improving the hydophilicity of the lens and modification of the surface roughness.

Surface roughness can be controlled by chemical and physical modifications. According to Yamakawa et al. (2003) not only the surface hydrophilicity but also the surface roughness has effect on the cell adhesion. They have observed that cell adhesion increases by increasing the surface roughness. Surface polishing, which is a common industrial technique, may improve the protein and cell repellence.

The other way to improve surface properties is through chemical modification.

One of the polymers to be grafted onto the lens surface includes poly(ethylene glycol) (PEG), because of its non-toxicity, non-immunogenicity and non-antigenic properties and it is known to reduce the protein adsorption because of high mobility in the hydrated state and steric hindrance. PEG grafting onto a variety of substrates has been reported to have a satisfactory effect as a protein-repellent agent. It may be predicted that the protein repellent properties of PEG would prevent the formation of extracellular matrix and thus cells adherence. Moreover, except this protective role, PEG is optically transparent at hydrated state which is of the greatest importance for materials to be used as optical devices (Bozukova 2007).

This idea was employed in a work conducted on lenses consisting of poly(HEMA-*co*-MMA). For this purpose, R-methoxy-PEG (mPEG) was reacted with the lens surface pre-modified by isocyanate groups. mPEGs with three different molar masses (1100, 2000, and 5000 g/mol) were tested to evaluate the effect of PEG chain length on the coating properties. It is concluded that the surface should be more hydrophilic when the mPEG chains are longer (Bozukova 2007).

Ekblad T et al. have reported that resistance to protein adsorption is greatly improved by grafting of short or long PEG chains on the surface of materials. Based on neutron reflectivity experiments, water absorption by short chain (oligo-ethylene glycol (OEG)) is responsible for reduced proteins absorption. In the case of longer PEG chains, it seems that a combination of steric hindrance and water-induced proteins resistant work together (Ekblad 2010).

Therefore, surface absorption of eye tear on contact lenses is very complex and depends on surface and bulk properties, such as water content, hydrophobicity, surface charge, surface roughness, wearing time and size and charge of the protein (Jones et al. 2003).

The biggest disadvantage of PEG is its weak chemical stability. The polymer is easily degraded through oxidative degradation, especially at high temperatures. Also, a range of bacteria can metabolize PEG chains with the help of alcohol dehydrogenase enzymes. The studies have shown that PEG coatings are not resistant and not suitable for prolonged exposure to proteins (Herold et al. 1989).

The purpose of this study was the preparation of semi-rigid lenses based on acrylic monomers by free radical polymerization. Here, acrylate functionalized PEG chains (PEGDA) were introduced into the lens bulk structure to overcome weak chemical stability of PEG on the surface of the lenses, while maintaining the protein repellant character. Two poly(ethylene glycol) (PEG) monomers with different molecular weights of 200 and 600 g/mol were reacted with acrylic acid by esterification. In this research different ratios of PEGDA and tri(ethylene glycol di methacrylate (TEGDMA) were adopted as crosslinking agents. The properties of the resulting lens materials including oxygen permeability, suitable physical and mechanical properties, roughness and bio-film formation were studied extensively as a function of the amount and molecular weight of PEGDA.

## Experimental

### Materials

Hydroxyethyl methacrylate (HEMA), methyl methacrylate (MMA*)* and acrylic acid were obtained from Merck, triethylene glycol dimethacrylate (TEGDMA) were provided from Evonik (Germany), dimethyl itaconate (DMI), poly (ethylene glycol) (PEG 200, 600), azobisisobutyronitrile **(**AIBN), 3-(trimethoxysilyl) propyl dimethacrylate (TMSPMA) were purchased from Sigma-Aldrich (Germany). NaHCO_3_, sulfuric acid, toluene and hydroquinone were obtained from Merck (Germany).

### Synthesis of poly(ethylene glycol) diacrylate (PEGDA)

Poly(ethylene glycol) diacrylate as polymeric monomers with two different molecular weights was synthesized through the esterification reaction. The esterification reaction was carried out at 84 °C in an oil bath using a Dean and Stark system. The values necessary for the esterification are presented in Table 1.

**Table 1:** Specific amounts of materials in poly (ethylene glycol diacrylate 200, 600) esterification

Material	Weight	Material	Weight
PEGDA 200	10 g	PEGDA 600	20 g
Acrylic acid	7.2 g	Acrylic acid	5.4 g
Toluene*	57.3 g	Toluene*	68 g
Hydroquinone**	1%	Hydroquinone**	1%
Sulfuric acid	4-8%	Sulfuric acid	4-8%

*The amount of toluene: 30% of the total components

**The amount of hydroquinone: 1% by weight of monomers

The reaction began to prevent the possibility of polymerization of acrylic acid at ambient temperature. First, the poly(ethylene glycol) was introduced into the reaction vessel, and then hydroquinone, an inhibitor weighed to the desired amount, was added into the reaction vessel. This was followed by addition of acrylic acid into the reaction mixture and then one-third of the initial amount of toluene (Table 1), along with sulfuric acid as a catalyst, were added into the mixture. With the gradual increase in temperature, the remaining toluene was added into the vessel and the reaction was continued for 24 h. After the desired time, the washing operation was performed using a 2% NaHCO_3_ solution and the separation of the aqueous and organic phase by a separator hopper. An organic phase containing PEGDA 200 and 600 was poured into the human body. The material was dried to dry matter and toluene was removed for 48 h in a vacuum oven at 45 °C with a yield of about 55.7%.

### Characterization of PEG diacrylate

#### FTIR spectroscopy

FTIR analysis was performed on a Bruker Equinox FTIR spectrometer (Germany) equipped with a Golden Gate single reflection ATR-FTIR attachment (attenuated total reflection) accessory. The resolution for all the infrared spectra was 4 cm^−1^, and there were 16 scans for each spectrum. Polymeric sheets were used in this study.

#### NMR spectroscopy

^1^H NMR spectra were recorded for PEGDA 200 and PEGDA 600 using deuterated chloroform as a solvent on a Bruker, 500 MHZ.

#### Molecular weight calculation of the poly(ethylene glycol) diacrylate (PEGDA)

Proton signals of the end group at chemical shifts of 5.8, 6.2 and 6.6 ppm are obtained by:

1 $${\text{Integral}}\; {\text{per}}\; {\text{proton}} = \frac{{{\text{Sum}}\;{\text{of}}\; {\text{the}}\; {\text{vinyl}}\; {\text{proton}}\; {\text{integral}}}}{{\# {\text{of}}\; {\text{proton}}\; {\text{in}}\; {\text{two}}\; {\text{vinyl }}\;{\text{end }}\;{\text{group}}}}$$

Based on Eq. (1), the result was 1.19 per proton for PEGDA 200 and 0.78 for PEGDA 600.

Calculate the number of repetitive monomer units:

2 $$n = \frac{{{\raise0.7ex\hbox{${{\text{Sum }}\;{\text{of}}\;{\text{methylene}}\;{\text{proton}}\;{\text{integral}}}$} \!\mathord{\left/ {\vphantom {{{\text{Sum }}\;{\text{of}}\;{\text{methylene}}\;{\text{proton}}\;{\text{integral}}} {\# {\text{of}}\;{\text{methylene}}\;{\text{protons}}}}}\right.\kern-0pt} \!\lower0.7ex\hbox{${\# {\text{of}}\;{\text{methylene}}\;{\text{protons}}}$}}}}{{{\text{Previously}}\;{\text{calculated}}\;{\text{integral}}\;{\text{per}}\;{\text{proton }}\;{\text{value}}}}$$

*The position of the proton signal OCH_2_CH_2_ (at chemical shifts of 3.6, 3.6 and 3.4 ppm).

According to Eq. (2), the number of repeating monomers in PEGDA 200 and PEGDA 600 was calculated to be 5.67 and 12.89.

Therefore, the M_n_ are:

$$M_{\text{n}} = \left( {55.06 + 71.06} \right) + \left( {44.05} \right)\left( {5.67} \right) = 376.4235 \;{\raise0.7ex\hbox{${\text{g}}$} \!\mathord{\left/ {\vphantom {{\text{g}} {\text{mol}}}}\right.\kern-0pt} \!\lower0.7ex\hbox{${\text{mol}}$}}\; \left( {{\text{PEGDA }}200} \right),$$

$$M_{\text{n}} = \left( {55.06 + 71.06} \right) + \left( {44.05} \right)\left( {12.89} \right) = 694.4645 \;{\raise0.7ex\hbox{${\text{g}}$} \!\mathord{\left/ {\vphantom {{\text{g}} {\text{mol}}}}\right.\kern-0pt} \!\lower0.7ex\hbox{${\text{mol}}$}}\; ({\text{PEGDA }}600).$$

#### Synthesis of PEGDA modified silicone-based diacrylate

At first, the desired components according to the formulation in Table 2 were placed in glass containers, and the reaction mixture was permitted at 23 °C (room temperature) using a magnetic stirrer for 45 min under constant *N*_2_ atmosphere. The weight of each batch was about 4 g. The homogeneous mixture was then poured into polyethylene molds, covered around with Teflon and paraffin tapes to prevent oxygen contacts on compound produced by polymerization process, and the dishes were sealed. Finally, the resulting compound was placed in the bath to allow further reaction. After transferring the components into the mold, in the first stage of polymerization, the compounds were cured at 70 °C for 2 h and then post-cured for further 48 h at 50 °C to complete curing of the materials in the polymerization process. To examine the state of the lenses, they were removed from the mold and kept for 24 h in order to complete the curing and polymerization operations inside the oven at 50 °C.

**Table 2:** Specific amounts of acrylate-based polymeric monomers in lens formulations

TEGDMA/PEGDA ratio (wt%)	100/0	80/20	50/50	20/80	0/100
TEGDMA (g)	0.29	0.23	0.14	0.06	–
PEGDA 200 (g)	–	0.07	0.19	0.30	0.38
PEGDA 600 (g)	–	0.14	0.35	0.56	0.70
HEMA (g)	0.15	0.15	0.15	0.15	0.15
TMSPMA (g)	1.00	1.00	1.00	1.00	1.00
MMA (g)	1.60	1.60	1.60	1.60	1.60
DMI (g)	1.00	1.00	1.00	1.00	1.00
AIBN (g)	0.02	0.02	0.02	0.02	0.02

### Characterization of a modified silicone-based diacrylate

#### Degree of conversion evaluation by FTIR spectrometry

FTIR has been used to identify the existence of functional groups and the determination of the numbers taken part in the reaction. To calculate the degree of conversion, the surface peak areas of the samples: before curing, and then curing for 1 h at 70 °C, 2 h at 70 °C, 24 h at 50 °C and 72 h at 50 °C were measured. The degree of absorption (surface peak area) of the aliphatic binary bond of C=C (in 1636 cm^−1^) and the highly adsorbed (surface peak area) C–O carbon–carbon functional group (1720 cm^−1^) as a reference peak before and after polymerization of each batch was obtained. The gradient was calculated using Eq. (3):

3 $${\text{Degree of conversion}} = \left( {1 - \frac{{\frac{{1636\;{\text{cm}}^{ - 1} }}{{1720\;{\text{cm}}^{ - 1} }}{\text{surface}}\;{\text{area}}\;{\text{at}}\;{\text{curing}}\;{\text{time}}}}{{\frac{{1636\;{\text{cm}}^{ - 1} }}{{1720\;{\text{cm}}^{ - 1} }}{\text{surface}}\;{\text{area}}\;{\text{before}}\;{\text{curing}}}}} \right) \times 100.$$

#### Water content

According to ISO 18369-4, the water content of the lenses was measured by weighing method. In this standard, the water content was defined as the percentage of water weights in a lens, which was completely saturated with standard salt solution (0.9%) at room temperature, and was calculated in accordance with Eq. (4):

4 $${\text{\% water content }} = \frac{{ {\text{weight of lens which is absorbed water solution - weight of dried lens }}}}{{ {\text{weight of lens which is absorbed water solution}}}}.$$

The specimens were weighed every 24 h for the first 2 days and then once every 48 h.

#### Oxygen permeability

Oxygen permeability increased logarithmically by increasing the amount of water content. This was achieved by the amount of equilibrium water content (EWC) in conventional hydrogels, and occurred when oxygen was allowed through the passage of water. The relationship between equilibrium water content and oxygen permeability is defined in Eq. (5) (Imani et al. 2007).

5 $$Dk = 1.67 e^{{0.0397{\text{EWC}}}}$$

#### Toxicity and biocompatibility

Cytotoxicity is known as the first test to assess the biocompatibility of a biomaterial. This test is based on ISO 10993 standard.

##### Negative control material

It is said that when the test is based on a standard protocol, it does not cause cytotoxicity. Basically, polyethylene, or polyethylene with high molecular weight, is used as a negative control material. For ceramic materials, aluminum oxide is commonly used as a negative control.

##### Positive control material

Substances which, when tested in accordance with the standard, it may produce a repeatable toxicity response. Substances such as dilute phenol and PVC solvents that are stable with tin can be used as a positive control.

##### Reagent controller

The extract is obtained from extraction conditions and laboratory methods, which can be either PBS or water.

### Cytotoxicity

Determination of cytotoxicity is done quantitatively and qualitatively:

(A) Quality evaluation: cells were examined by reverse phase optical microscopy. Finally, the status of the samples is described descriptively.

(B) Quantitative evaluation: This method can be used to measure cases such as cell death, cell growth prevention and proliferation, or create colony, number of cells, protein content, enzymes release, release of vital color, discoloration, etc. The full confidence may be achieved on the selected evaluation methods. Because test materials would be unreliable if the tested materials interfere with the testing or measurement system.

### Cell culture

L929 fibroblast cell was used for cell culture. This cell is from the National Bank of Iran (NCBI) at the Pasteur Institute of Iran. The sterilized samples with 1 cm^2^ area were placed in the center of 24 well plates and then approximately 5 × 10^4^ cells per cm^2^ in 1640 RPMI medium with 10–20% FBS were added. The plate was left in a 37 °C incubator with 5% CO_2_ for 24 h. According to the standard, after 24 h, the supernatant was removed and the cellular layers were washed with PBS. The cultured cells were regularly examined by microscopy.

### Determination of the percentage of live suspension cells and cell count

The live-cell membrane does not allow the ingestion of non-electrolyte dye into the cell. This property was used to count live cells (in a culture medium flask).

For this purpose one drop of cell suspension (approximately 2 million cells per milliliter) was mixed with a trypan blue drop, and after 1–2 min, the percentage of live cells (non-colored cells) and dead cells (cells colored beams are counted by optical microscope, and the ratio of live cells can be calculated from the relationship of Eq. (6).

6 $${\text{Percentage of live cells}} = \frac{\text{Number of live cells}}{\text{Total living cells counted}}$$

The samples were sterilized by ethanol at temperatures of 70 °C before cytotoxicity and biocompatibility testing.

### Protein absorption test

Several microscopic, photometric and imaging methods are employed to investigate the absorption of proteins on surfaces of contact lenses. Microscopic and imaging methods do not display good accuracy for quantitative measurement purposes. Different biochemical methods such as enzyme-linked immunosorbent assay (ELISA) are very useful due to increase sensitivity, detection accuracy of specific proteins. ELISA is the most widely used method for studying tear film proteins deposition. ELISA is based on the identification of the antibodies in proteins solution, which uses fluorescence colorimetry or chemical correction to determine the amount of protein absorbed. In this study, BCA protein kit (contain lysozyme and albumin) was employed to determine the amount of sediment protein on the lens surface.

### Atomic force microscopy (AFM)

Non-contact mode atomic force microscopy, Dual Scope Co (DME), 25-200E model: DS with 10 nm radius silicon probe and field strength of 150–190 kHz, were used. The roughness was calculated by SPM-DME software. To study the roughness of the surface, 24 mm diameter lens samples were synthesized into the polyethylene molds.

### Scanning electron microscopy (SEM)

Scanning electron microscopy was used to study surface lens morphology. SEM micrographs were obtained using a VEGA (TSCAN, CHEC) scanning electron microscope. Samples were mounted on aluminum mounts and coated with gold (15 nm) using a Techniques Hummer II sputter coater.

### Contact angle

Contact angles were measured with de-ionized water on a contact lens sheet at room temperature. The water drop was placed on the lens surface by an insulin syringe. Contact angles were measured by “Image Analyzer Software” on images acquired by a camera. The test was carried out on 5 sheets and 4 drops of water were applied on each sheet.

### Hardness

Hardness is one of the criteria for the strength of solids against plastic deformation. The hardness of lenses was measured by durometer for measuring the shore-D hardness of polymers in accordance with the ISO 868 standard. Each disc-shape sample was evaluated by three different points, and the average hardness and standard deviation of these points was declared as the final result.

## Results and discussion

### Poly (ethylene glycol) diacrylate characterization

Poly(ethylene glycol) diacrylate (PEGDA) is a poly(ethylene glycol) derivative that has unique properties such as hydrophilicity, flexibility, non-toxicity and non-immunogenecity, and contains acrylic groups on both ends of its chain, which can take part in the polymerization process. Therefore, poly(ethylene glycol) diacrylates can form chemical gels alone or in combination with other polymer monomers linked by covalent bonding through networking reactions.

**Fig. 1:**
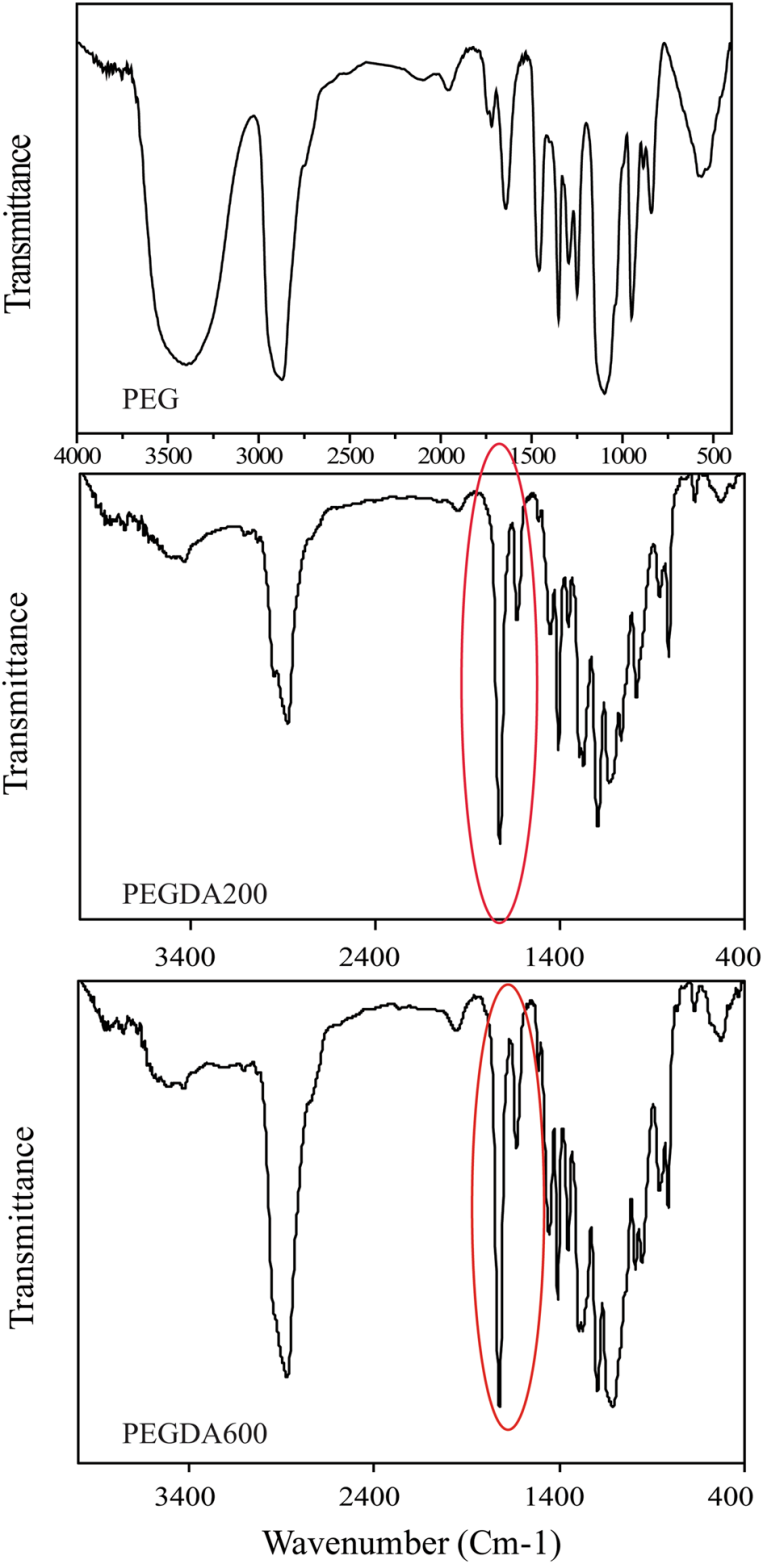
FTIR spectrum of PEG, PEGDA 200 and PEGDA 600

Poly(ethylene glycol) diacrylate (PEGDA) was synthesized by esterification of poly (ethylene glycol(200 and 600 with acrylic acid. Fourier transform infrared spectroscopy (FTIR) was used to analyze the chemical composition of acrylated poly (ethylene glycol). FTIR spectra of poly (ethylene glycol), PEGDA 200 and 600 are shown in Fig. 1.

According to Fig. 1, the absorption band at 1633 cm^−1^ may be attributed to a C=C aliphatic double bond and a peak at wavelength of 1724 cm^−1^ which corresponds to carbonyl group (C=O). The broad absorption peak of OH is assigned to poly (ethylene glycol) at 3500–3400 cm^−1^ and it is almost disappeared in PEGDA (Imani et al. 2007). The presence of two characteristic peaks of C=O and C=C bonds and the disappearance of a hydroxyl peak may confirm the addition of acrylate group to poly (ethylene glycol).

**Fig. 2:**
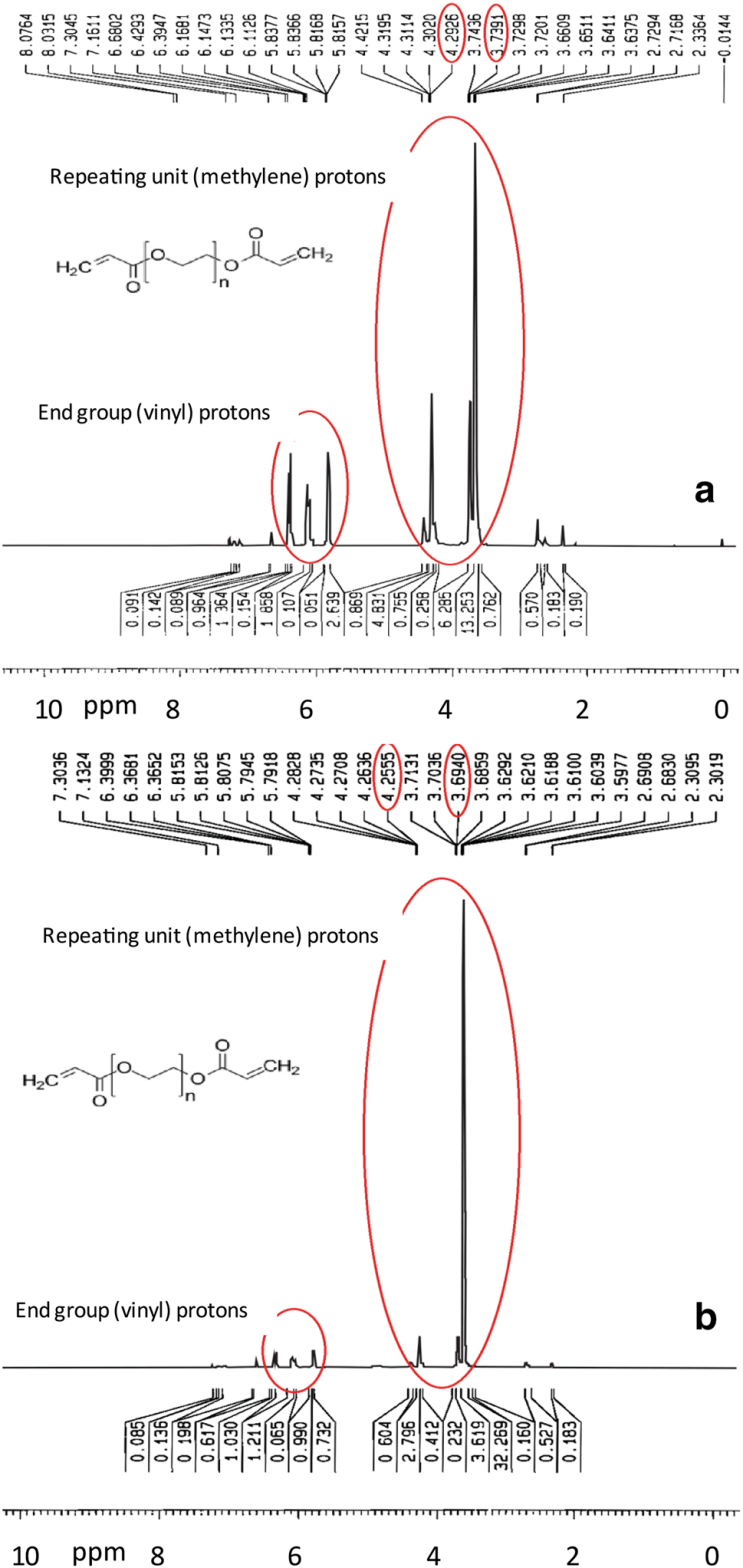
^1^H NMR spectra of **a** PEGDA 200 and **b** PEGDA 600

Figure 2 shows the ^1^H NMR patterns of synthesized PEGDA 200, 600 prepared in deuterated chloroform.

The ^1^H NMR results show that chemical shifts in the range of 5.8, 6.2 and 6.4 ppm in PEGDA 200 and 600 indicate that the reaction was successfully performed (Imani et al. 2007). As well as the chemical shift of proton resonance detected in the range of 3 and 4 ppm, and the chemical shift between 4 and 5 ppm is related to the OH-groups, which indicates that acrylic acid has participated in the esterification reaction with poly (ethylene glycol). It is obvious that the peak of poly (ethylene glycol) appears at 3.74 ppm resonance. Also, in PEGDA 200 its resonant peak at 4.29 ppm is sharp with higher intensity compared to PEGDA 600 resonance peak at approximately 4.25 ppm, due to both substances of different molecular weights.

### Degree of conversion

In this study, the polymeric monomers (PEGDA 200 and 600) were synthesized by free radical polymerization.

The degree of conversion of formulated lenses was determined on taking 3 samples: TEGDMA as control, TEGDMA/PEGDA 200: 50/50, TEGDMA/PEGDA 600: 50/50 compositions (Table 2).

**Fig. 3:**
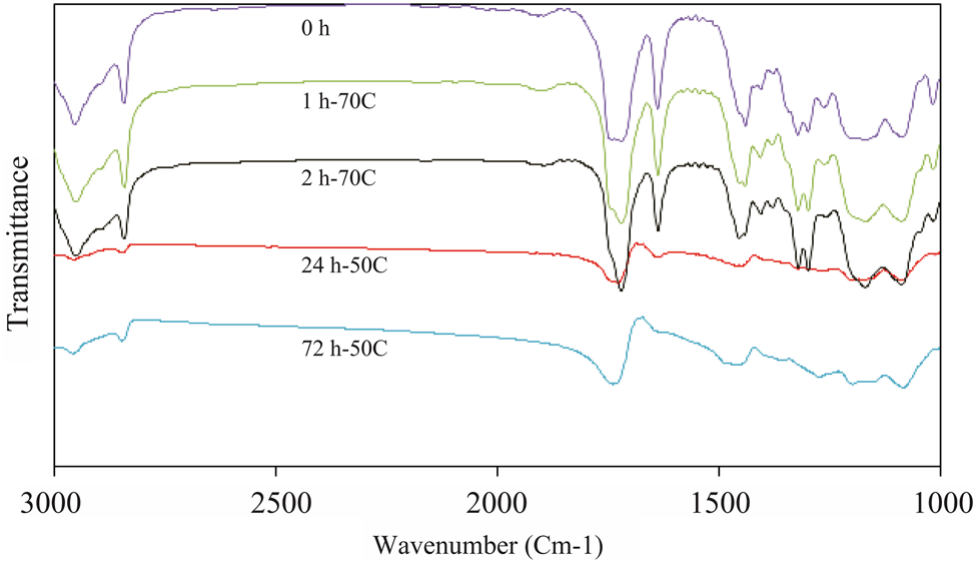
FTIR spectra of control lens, cured based on TEGDMA as crosslinker, at 50 and 70 °C and different curing times

FTIR spectroscopy was performed on all 3 specimens: before curing, cured at 70 °C for 1 and 2 h, and followed by post-curing at 50 °C for 24 and 72 h; and then the degree of conversion was studied. The spectrum of control lens cured based on TEGDMA as crosslinker at different curing times is illustrated in Fig. 3.

**Fig. 4:**
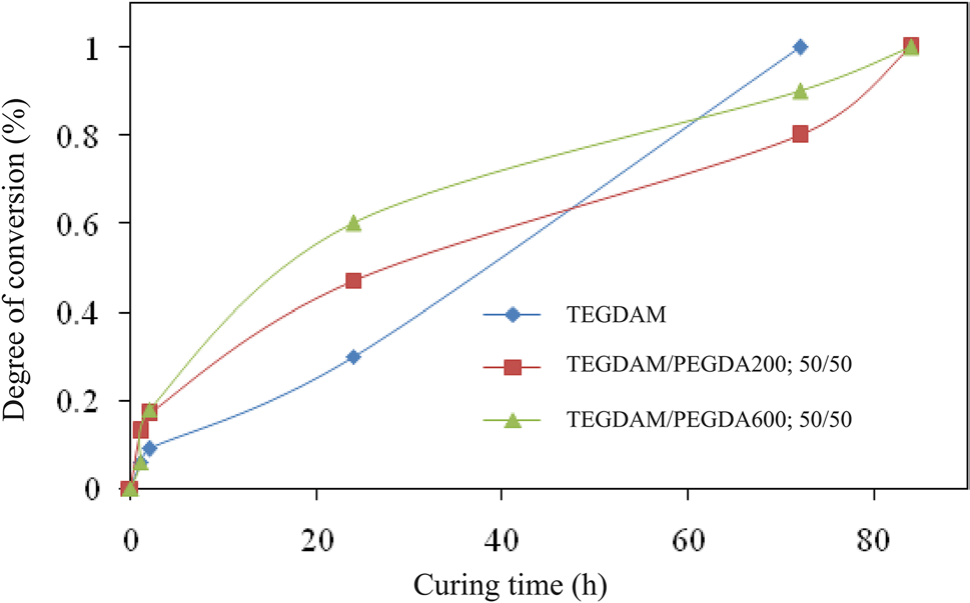
Degree of conversion curve at different curing times for 50/50 ratio of TEGDMA/PEGDA

The degree of conversion at different curing times and temperatures originated from the FTIR spectra are presented in Fig. 4. Three compounds including TEGDMA, TEGDMA/PEGDA 200: 50/50 and TEGDMA/PEGDA 600: 50/50 from Table 2 are selected.

The results of conversion measurement show that increasing the curing time and post-curing of the lenses leads to an increased degree of conversion until the reaction is completed.

According to our previous report (Hanifeh et al. 2018), it was concluded that although the increase in temperature would be a good approach to achieve 100% degree of conversion, the samples prepared above 70 °C show many defects and weak mechanical properties.

This could be due to increases in temperature during the manufacturing process which causes thermal stress in the mold and produces pressure which creates cracks in the cured samples. By increasing the reaction rate, the amount of generated heat would be greater and, therefore, the cracks are spread further. Therefore, the optimum temperature for the first curing is found to be 70 °C and the post-curing temperature is 50 °C and the perfect time to complete the reaction could be 72 h (Hanifeh et al. 2018). As shown in IR spectra, the control sample (Fig. 3), with increasing the curing time, the carbon–carbon double bond in the range of 1636 cm^−1^ is shorter and it finally disappears.

It should also be noted that, as shown in Fig. 4, two samples containing poly (ethylene glycol) dicarylated (PEGDA 200 and 600),), at 50/50 have not reached the degree of curing conversion of 100% over a period of 72 h compared with the control sample. This may be due to the formulation of the lens; 3 formulations such as: PEGDA 200 and PEGDA 600 and TEGDMA in different ratios were used in different compositions. Therefore, it can be concluded that 72 h time period for a lens containing poly (ethylene–glycol) diacrylate monomers is insufficient and it should be about 84 h to reach a complete conversion.

### Hardness

**Fig. 5:**
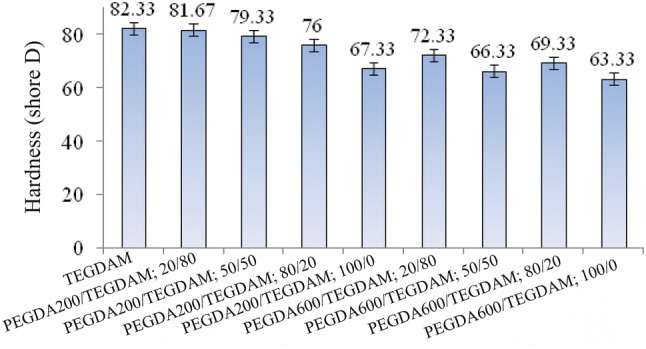
Hardness of the formulated lenses based on different composition ratios, TEGDMA, PEGDA 200 and PEGDA 600 according to Table 2

Hardness is one of the criteria for the resistance of materials to plastic flow. In this research, the hardness of the samples was measured using shore D scale durometer method. The average hardness of the disk-shape samples is shown in Fig. 5.

The crosslinking agent plays a key role to improve the crack resistance, stiffness, hardness and etc. of the final products. Ethylene glycol dimethacrylate and triethylene glycol dimethacrylate are commonly used as crosslinking agents. In this study, the effect of two poly (ethylene glycol) diacrylates with different molecular weights of 200 and 600 on hardness of the lenses was studied. According to the hardness diagram (Fig. 5), samples containing PEGDA 200 display higher hardness than PEGDA 600 specimens and are consistent with the results of previous studies. The flexibility of the polymer structures containing diacrylates and dimetacrylates, such as EGDMA, TEGDMA and PEGDA increases with increasing the length of ethoxy repeating groups (Arimal et al. 1995).

While the crosslinkers with longer chains rotate easily around its ether-linkage and are more flexible. Therefore, the presence of multiple ether-linkage improves flexibility, and thus allows more chain mobility in the structure resulting in reduced sample hardness (Arimal et al. 1995; Beatty et al. 1993; Mabilleau et al. 2005).

### Wettability and contact angle

**Fig. 6:**
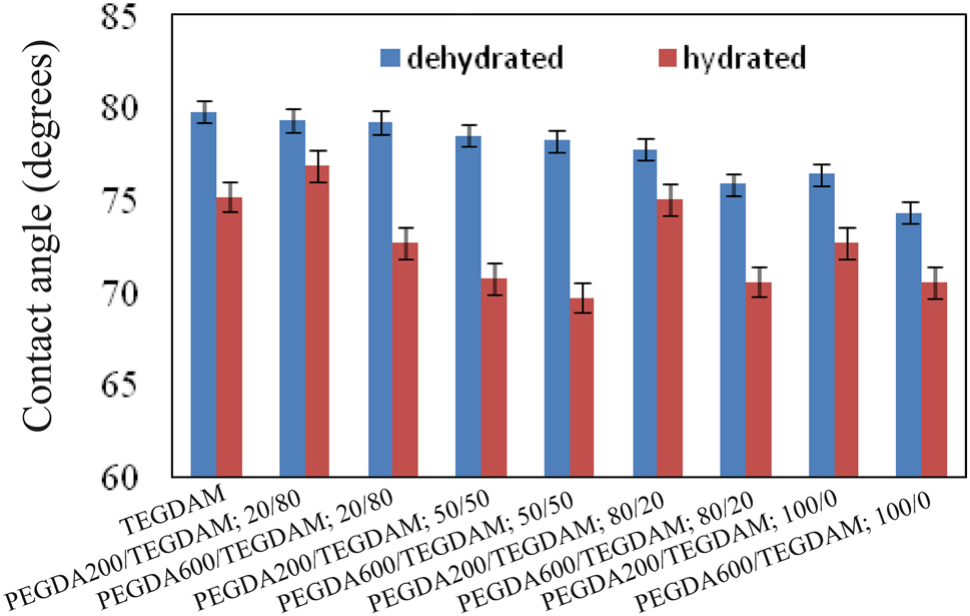
Contact angle values in dry and wet conditions for formulated lenses based on different composition ratios of TEGDMA, PEGDA 200 and PEGDA 600 according to Table 2

One of the most important features of contact lenses is their degree of hydrophilicity or hydrophobicity. When the lenses are not hydrophilic enough, by wearing the lenses the patient may feel discomfort. Hydrophobicity of the samples made according to Table 2; was measured by the sessile drop contact angle test and the mean values obtained from 5 contact angle measurements are shown in Fig. 6.

As it is shown in Fig. 6, with increasing the molecular weight in PEGDA 600, the length of hydrophobic backbone increases and the number of hydroxyl groups, based on their equal weight percent, decreases which leads to lower crosslinkling density and lower hydrophilicity which appear in both wet and dry conditions.

Also, the contact angle measured under wet condition is significantly lower than that in dry condition, because in wet condition lenses swell and more hydrophilic space is created.

Since contact angles of the wet samples are measured in air, the PEGDA and other hydrophilic chains in the lens surface tend to rearrange and hide inside the bulk of the lens to avoid the air hydrophobic environment. While the shorter chains (PEGDA 200), rearrange easier than longer chains (PEGDA 600), (Bozukova 2007). Therefore, the hydrophilicity of lenses containing PEGDA 600 is increased.

### Water absorption and water content

**Fig. 7:**
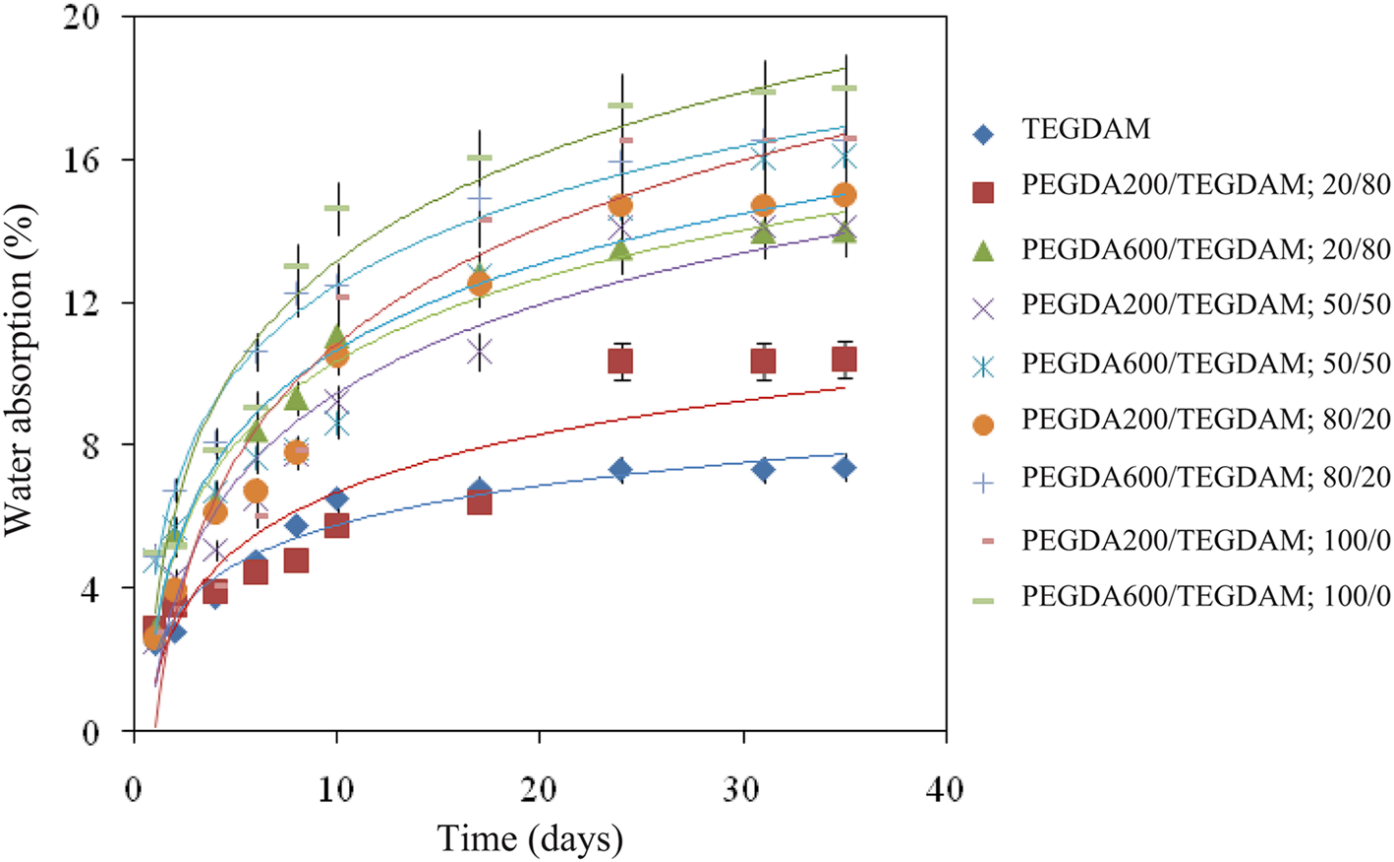
Water absorption rates in formulated lens based on different compositions ratios of TEGDMA, PEGDA 200 and PEGDA 600 according to Table 2

The water absorption diagram of the lenses, according to Table 2, is shown in Fig. 7. The chain effects of crosslinking agents (PEGDA 200 and 600) in relation to polymer characteristics are studied under wet condition. Hardness, stiffness, and Young modulus seem to be dependent on the chain length of the crosslinker (Mabilleau et al. 2005). In the current research, the amount of water absorption is affected by the crosslinking agent introduced into the polymer network. Lenses containing PEGDA 600 show higher water absorption than the specimens containing PEGDA 200.

Flory presented the first theory on swelling of crosslinked polymer gels based on the Gaussian distribution of polymer chains. This model describes the degree of equilibrium of the crosslinked polymer, in the form that the swelling rate is supposed to be due to the elastic forces of polymeric chain contraction and the thermodynamic compatibility of polymer and solvent (Mabilleau et al. 2005; Vidal-Rohr et al. 2018).

The hydrophilic properties of ethoxy groups in dimethacrylates such as EGDMA, TEGDMA and PEGDA are due to the presence of oxygen in forming hydrogen bonding, following interactions between water molecules and polymer segments,. Therefore, it is expected that the water-polymer tendency increases with the increase in the number of ethoxy repeating groups (Arimal et al. 1995), which is confirmed by water absorption and water content results of the present study based on the following trend: TEGDMA < PEGDA 200 < PEGDA 600.

### Relative oxygen permeability

Oxygen permeability increases logarithmically by increasing the amount of water content.

This is achieved by the amount of equilibrium water content in conventional hydrogels and occurs when oxygen is able to pass water. The relation between equilibrium water content and oxygen permeability is defined in Eq. (5) (Imani et al. 2007).

Therefore, in accordance with Eq. (3) and using the water content data, oxygen permeability of the prepared lenses was obtained in accordance with Table 4.

As it is obvious in Table 3, increasing the percentage of PEGDA in the lens formulations the amount of water content and the permeability of oxygen increase, which is in agreement with the results of previous experiments. The presence of PEGDA with a molecular weight of 600 and long-chain lengths increase hydrophilicity; consequently increase the water content of the lenses and permeation of the oxygen.

**Table 3:** Oxygen permeability rates in formulated lens based on different composition ratios of TEGDMA, PEGDA 200 and PEGDA 600 (Table 2)

Sample	Equilibrium water content, EWC (%)	Relative oxygen permeability (*Dk*)
TEGDMA	7.3	2.2
PEGDA 200/TEGDMA: 20/80	10.4	2.5
PEGDA 600/TEGDMA: 20/80	13.5	2.8
PEGDA 200/TEGDMA: 50/50	14.1	2.9
PEGDA 600/TEGDMA: 50/50	14.6	2.9
PEGDA 200/TEGDMA: 80/20	14.7	2.9
PEGDA 600/TEGDMA: 80/20	15.9	3.1
PEGDA 200/TEGDMA: 100/0	16.5	3.2
PEGDA600/TEGDMA: 100/0	17.5	3.4

### Biocompatibility

**Fig. 8:**
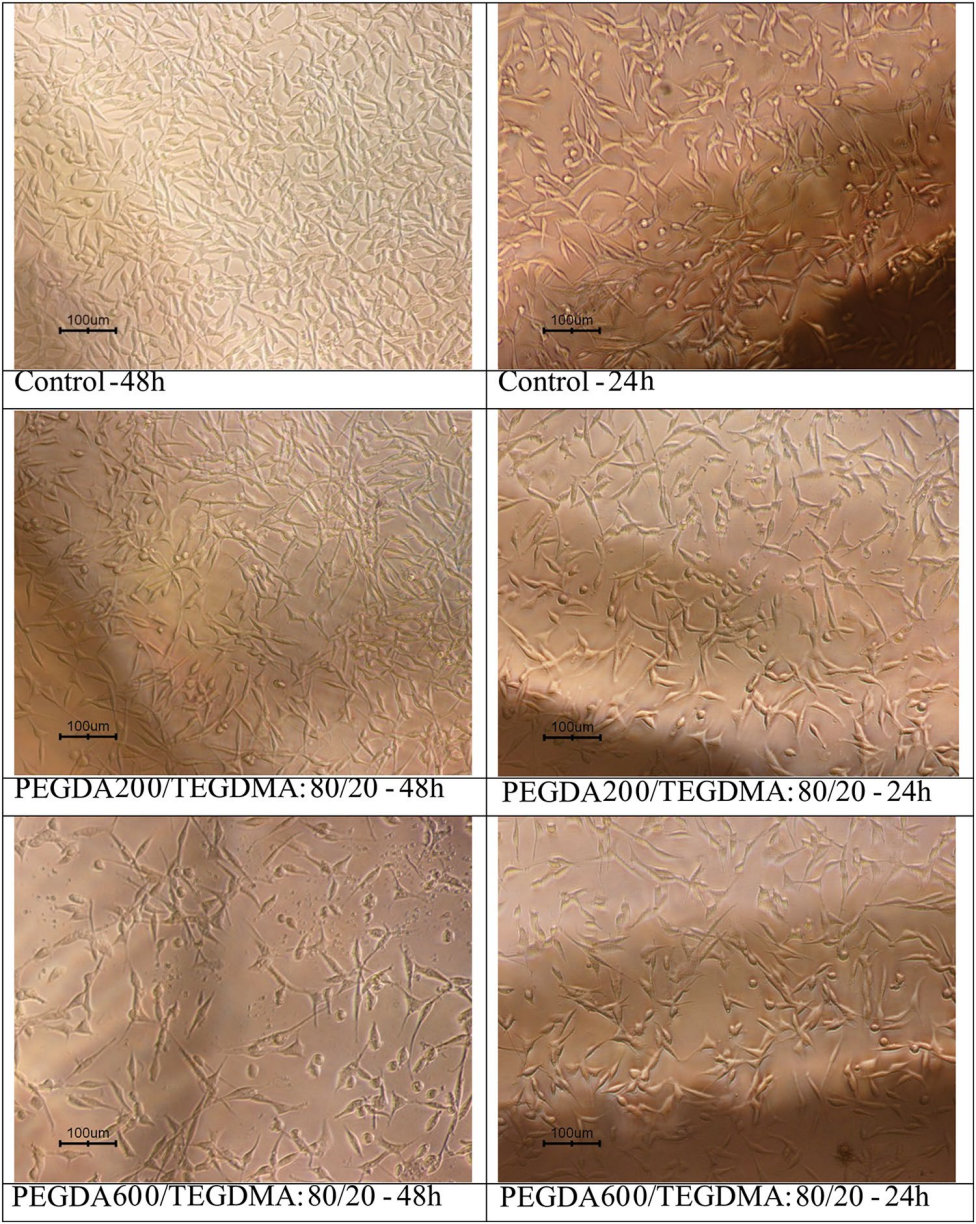
The results of toxicity test for the tissue culture plate (control), PEGDA200/TEGDAM: 80/20 and PEGDA600/TEGDAM: 80/20 according to Table 2 for 24 and 48 h

The cytotoxicity tests were performed according to ISO 10993, using indirect contact method by culturing the L929 mouse fibroblasts. In this research 9 samples were prepared according to Tables 1 and 2 and incubated in culture media solution at 37 °C for 7 days. The extract dilution exposure method, applied on a wide variety of medical devices, was performed to detect toxins leached out from the exposed surfaces. The L929 cells were placed in the extracted solution for 24 h and 48 h, and the images from the optical microscopy showed that the cells were grown well in all the specimens; an indication that they were free of toxic pathogens and left no harmful effects on the cells. Figure 8 shows the cytotoxicity results of incubated tissue culture plate (control), PEGDA 200/TEGDAM: 80/20, PEGDA 600/TEGDAM: 80/20 according to Table 2 for 24 and 48 h.).

Biocompatibility is essentially dependent on the physical, chemical and morphological properties of the implants. Tamada and Ikada (1994) have reported that adhesion and cell growth depend on hydrophilicity and water content of the substrates.

They also showed that fibroblasts are very rapidly propagated on the substrate with contact angle of about 70°.

Another study shows that the cell adhesion can be influenced by free energy of the implant surfaces.

**Fig. 9:**
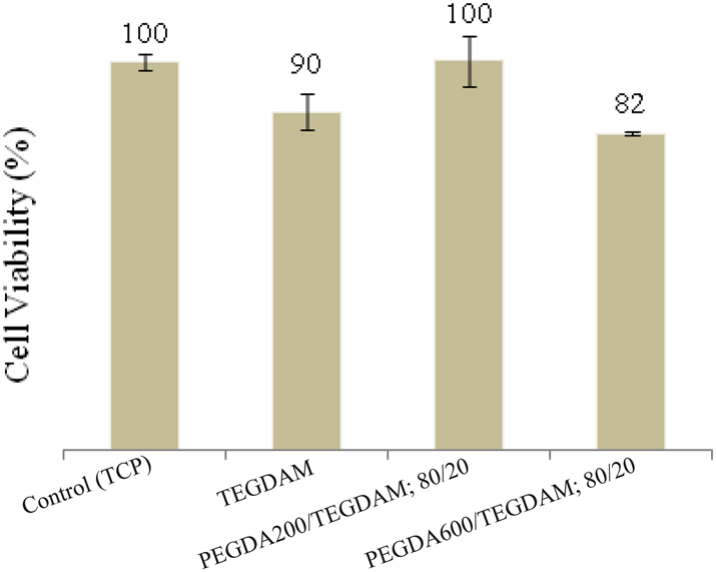
Cell viability of control, TEGDMA, PEGDA 200/TEGDAM: 80/20, PEGDA 600/TEGDAM: 80/20 after 24 h of cell culture

In subsequent studies, the effect of PEG grafting on a poly(methyl methacrylate) substrate has been investigated and it is suggested that PEG chains are bonded into poly(methyl methacrylate) substrate by covalent bonds which leads to irreversible chemical changes on the surface of the poly(ethyl methacrylate). PEG chains are grafted on the interface of poly(ethyl methacrylate). This configuration can change the high surface-tension of a system (water/polymethyl methacrylate) to a lower surface-tension (water/PEG/poly(methyl methacrylate)). The end group of PEG chains is exposed on the surface, and the other side of chains moves freely. The free end of the PEG chains undergoes a fixed rearrangement. For example, when the PEG is in contact with aqueous solution, the hydrophilic side of chain is active, however, when the PEG is in contact with a hydrophobic environment such as air, the hydrophilic parts of the chain would be hidden (Tamada and Ikada 1994; Kim and Wee 2001; Cunanan et al. 1991). The L929 cells viability of the samples was also quantitatively measured and the results of the control (tissue culture plate), TEGDAM, PEGDA 200/TEGDAM (20/80) and PEGDA 600/TEGDAM (20/80), after 24 h in accordance with Tables 1 and 2 are shown in Fig. 9. As can be seen from the results, the results of the optical microscopy, which qualitatively reports the cytotoxicity, confirms MTT assay.

The presence of PEGDA in the lens formulation has led to acquire more hydrophilicity and helps to reduce cell surface adhesion. Therefore, after cell fixation on the samples after 48 h of contact with L929 cell, the effect of cell adhesion was studied by scanning electron microscopy.

**Fig. 10:**
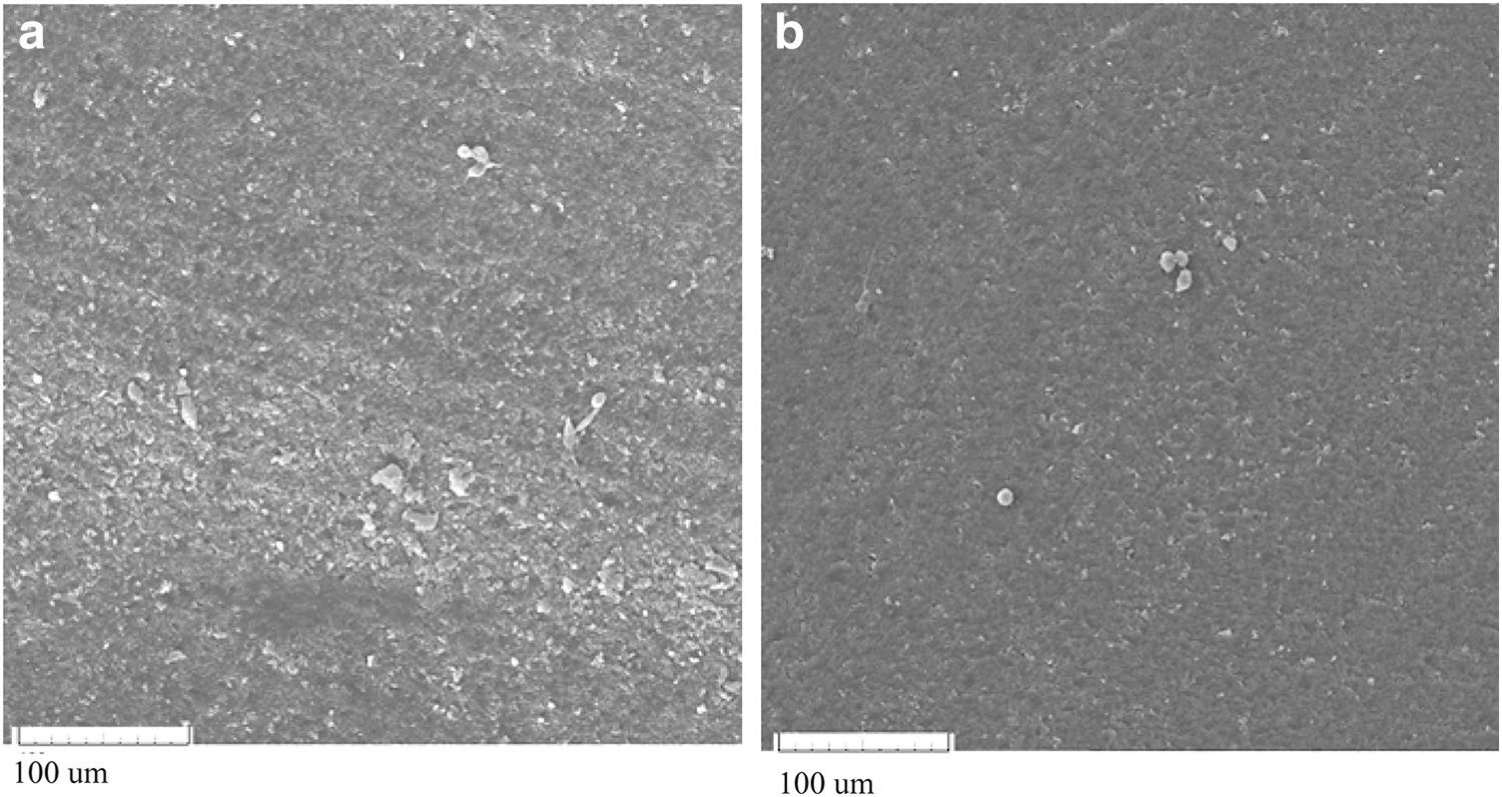
SEM micrograms of fixed cell: **a** control, **b** PEGDA 200/TEGDAM: 50/50, after 48 h cell culture

As shown in Fig. 10, cellular non-adhesion on the samples can be verified.

Surface hydrophilicity is one of the approaches that can affect cell adhesion properties. Recent research describes a system that considers interface energies (such as interface energies of cells/substrate, interface energies of the cell/liquid, and interface energies of liquid/solid). Another research also refers to that of the other factors to be considered such as surface roughness, crystalline/amorphous surface, and surface chain mobility in different media (Kim and Wee 2001; Cunanan et al. 1991; Schakenraad et al. 1986).

### Surface roughness

**Fig. 11:**
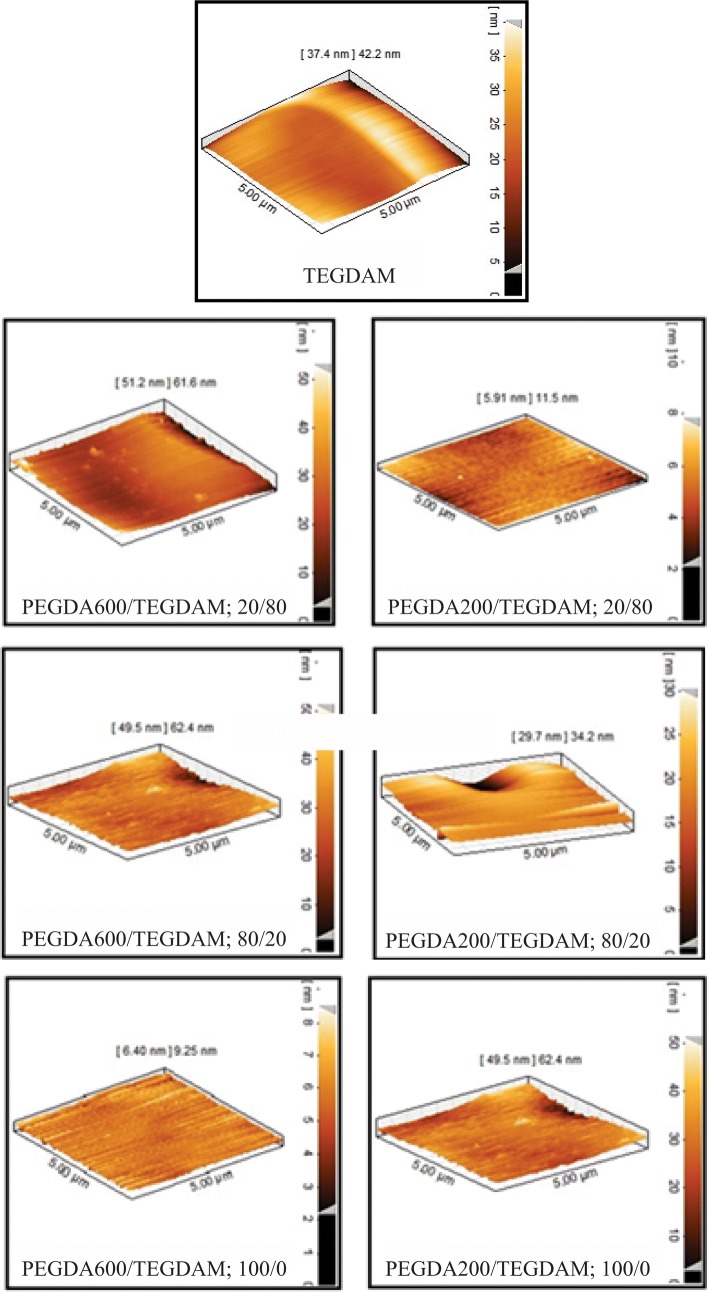
AFM study on different composition ratios of TEGDMA, PEGDA 200 and PEGDA 600 according to Table 2

Poly (ethylene glycol) coatings among various biocompatible polymers have been considered as a polymer influence in reducing protein uptake and cell adhesion on different substrates. The anti-fouling properties of poly(ethylene glycol) are mainly due to their high chains mobility, high free volume and steric hindrance. In particular, PEG chains have hydrophilic repeating units, flexible conformation, and even some branched structure derivatives associated with steric hindrance (Jee and Kim 2015). Using poly(ethylene glycol) in formulation provides lenses with higher hydophilicity and anti-fouling properties. The intact surface of the prepared lenses was investigated by atomic force microscopy to investigate the surface roughness. According to Table 4, in which the values of the roughness parameters are measured for each sample and Fig. 11 shows that almost the surface roughness of all lenses containing poly(ethylene glycol) is approximately the same in their formulation.

**Table 4:** The values of roughness parameters, *S*_a_ (average roughness) and *S*_q_ (the root mean square) in formulated lens based on different composition ratios of TEGDMA, PEGDA 200 and PEGDA 600 according to Table 2

Sample	5 × 5 μm^2^	
	*S*_a_ (nm)	*S*_q_ (nm)
TEGDMA	3.98 ± 0.61	4.90 ± 0.41
PEGDA 200/TEGDMA: 20/80	4.53 ± 0.47	6.46 ± 0.60
PEGDA 600/TEGDMA: 20/80	4.05 ± 0.64	5.15 ± 0.18
PEGDA 200/TEGDMA: 80/20	0.75 ± 2.76	4.14 ± 0.35
PEGDA 600/TEGDMA: 80/20	1.94 ± 0.76	2.79 ± 0.79
PEGDA 200/TEGDMA: 100/0	2.04 ± 0.32	3.17 ± 0.47
PEGDA 600/TEGDMA: 100/0	1.90 ± 0.76	2.64 ± 0.96

In fact, the surface of PEGDA chain lenses, due to hydophilicity, produce a smoother surface, thus reducing the surface roughness by increasing the percentage of PEGDA chains, and producing PEGDA 600 lenses with a higher level of smooth surface.

The effect of surface roughness on cell adhesion of contact lenses is still not fully understood. According to previous studies, surface roughness seems to be related to the formation of sediment and accumulation of the microorganisms on the surface. More surface roughness provides more surfaces to active sites and improves the thermodynamic reactions. The formation of a biofilm takes place in two stages: initial adhesion of the cells to the polymer surface, and then growth and proliferation of the cells to form multi-layered cellular branches and formation of glycocalyx (Giraldez et al. 2010). The relationship between roughness and wettability was defined by Wenzel (1936). A simple Venzel model was based on the assumption that rough surfaces increase the interface of solid/liquid compared to smooth surfaces. In this way, with increasing the surface roughness, the wettability of surface is improved (Elkins et al. 2014). It can be concluded that two factors of hydrophilicity and roughness are important parameters for adhesion to the lens. Hydrophobicity is considered as a dominant factor, while the roughness of this adhesion is of secondary importance. According to a research conducted by Yamakava and colleagues, not only surface hydrophilicity but also the roughness of the surface affects cell adhesion. Cell adhesion increases with increasing surface roughness (Yamakawa et al. 2003). Therefore, in this study, lenses were prepared with intact surfaces by atomic force microscopy.

In a research conducted by Zakkawa et al., the surface of lenses which were improved by chemical coating of poly(ethylene glycol) was observed through AFM. Based on the results, it was shown that the surface of the produced lenses is rough and that the bonding of single-layer poly(ethylene glycol) does not eliminate the roughness.

In fact, the bonding of poly(ethylene glycol) with a high molecular weight component, such as 1000 and 2000 causes rougher surfaces, while, homogeneity and surface smoothness improve by grafting with high molecular PEG (e.g., molecular weight of 5000). Also, the study also points to the presentence of poly(ethylene glycol) and increase of water absorption (Bozukova 2007).

### Protein absorption

Accumulation of protein on the lenses over time increases the microorganism adhesion and has a great effect on the health of the cornea and the lens comfort. Tear film contains anti-bacterial tear proteins (lysozyme, lactoferrin, and albumin) that play an effective role in preventing infection and inflammation in the eye. During the use of the lens, these elements accumulate on the surface of the lens and form a rich film of protein. The composition of this film depends on the chemical composition of the contact lenses.

The variation in the contact lens and lens storage solution can greatly affect the function of the epithelial layer. It is important to note that different lens storage solutions have different effects on the degree of cell adhesion on the lens surface (Elkins et al. 2014). Poly(ethylene glycol) or oligo (ethylene glycol) OEG is one of the most well-known anti-fouling materials. PEG or OEG modified surfaces inhibit protein absorption in a buffer solution. It is believed that the effect of steric hindrance and hydrophilic suppression to create surface resistance in protein absorption is one of the most important factors (Cho et al. 2007). Poly(ethylene glycol) diacrylate is a derivative of poly(ethylene glycol), which can be used to modify the polymer to reduce protein uptake and adhesion of blood platelets and biocompatible anti-deposition agent (Lin et al. 2014).

A 40% of the tear film proteins made of lysozyme, and it is deposited by about 36% and 95% depending on the type of ophthalmic lenses (Keith et al. 1997).

**Fig. 12:**
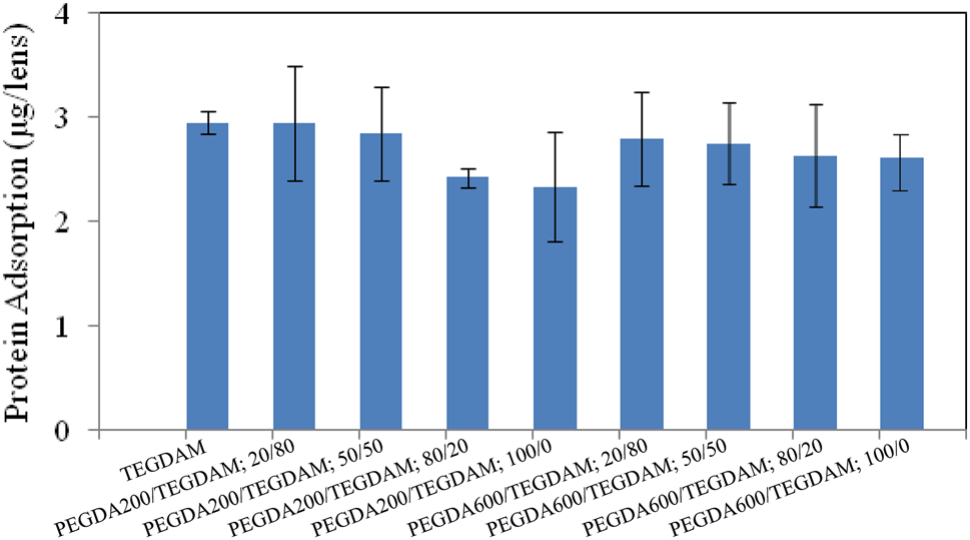
Amounts of absorbed protein on different composition ratios of TEGDMA, PEGDA 200 and PEGDA 600 according to Table 2

Figure 12 shows the amount of protein absorption for each lens. As can be seen, by increasing the poly(ethylene glycol) diacrylate monomer, the protein absorption amount is reduced.

Obviously, since poly(ethylene glycol) diacrylate 600 provides higher hydrophilicity than that of poly(ethylene glycol) diacrylate 200, so the protein absorption of the former would be lower.

However, no significant difference was recorded between the protein absorption of these samples (*p* > 0.05).

## Conclusion

In this research, the fabrication process of lens ophthalmic discs take place in two steps: in the first step, the ingredients are mixed in a glass container under the nitrogen atmosphere and transferred into a PE mold prior to pre-polymerization at 70 °C. Then, in the second stage, it is post-cured at 45 °C for 72 h. The hardness of the lenses is especially important in improving their chain flexibility. This parameter for lenses with poly(ethylene glycol) 200 monomer is in the range of (69.3 to 82.3) ± 0.6 shore D and for poly(ethylene glycol) 600 monomer it is in the range (63.3 to 79.3) ± 0.6 shore D. Also, the value of this parameter for the control lens, containing TEGDMA monomer only, is 82.3.6 ± 0.83 shore D.

The water content of the lenses is directly related to the oxygen permeation of the lens so that by increasing the water content, the oxygen permeability would increase. The water content of lenses with PEGDA 200 monomers is (10.4 to 16.5) ± 0.6 (%) and that of lenses with PEGDA 600 monomer is (13.5 to 17.5) ± 0.8 (%), with the water content of control lens is 7.3 ± 7 (%).

Surface roughness is associated with the formation of biofilm deposition and the agglomerated microorganisms on the surface. Due to the roughness of the surface of the lenses prepared in this project, the lenses containing PEGDA 600 exhibited a lesser roughness than lenses with a PEGDA of 200, which could also affect the absorption of protein. So, according to the results of the protein absorption investigation, a PEGDA 600 lens with higher hydrophilicity showed lower protein absorption.
